# Tension-Dominant Orthodontic Loading and Buccal Periodontal Phenotype Preservation: An Integrative Mechanobiological Model Supported by FEM and a Proof-of-Concept CBCT

**DOI:** 10.3390/jfb17010047

**Published:** 2026-01-16

**Authors:** Anna Ewa Kuc, Jacek Kotuła, Kamil Sybilski, Szymon Saternus, Jerzy Małachowski, Natalia Kuc, Grzegorz Hajduk, Joanna Lis, Beata Kawala, Michał Sarul, Magdalena Sulewska

**Affiliations:** 1 Department of Dentofacial Orthopedics and Orthodontics, Wroclaw Medical University, 50-425 Wroclaw, Poland; j_kotula@poczta.onet.pl (J.K.); joanna.lis@umw.edu.pl (J.L.); beata.kawala@umw.edu.pl (B.K.); 2 Faculty of Mechanical Engineering, Military University of Technology, 00-908 Warsaw, Poland; kamil.sybilski@wat.edu.pl (K.S.); szymon.saternus@wat.edu.pl (S.S.); jerzy.malachowski@wat.edu.pl (J.M.); 3 Faculty of Medicine, Medical University in Bialystok, ul. Kilińskiego 1, 15-089 Bialystok, Poland; nataliakuc.med@gmail.com; 4 Chair and Department of Oral Surgery, Medical University of Lublin, Witolda Chodźki 6 Street, 20-093 Lublin, Poland; esso53@wp.pl; 5 Department of Integrated Dentistry, Wroclaw Medical University, 50-425 Wroclaw, Poland; michal.sarul@umw.edu.pl; 6 Department of Periodontal and Oral Mucosa Diseases, Medical University in Bialystok, ul. Waszyngtona 13, 15-269 Bialystok, Poland; magdalena.sulewska@umb.edu.pl

**Keywords:** mechanobiology, buccal phenotype, tensile microstrain, finite element analysis, stress redistribution, periodontal ligament mechanics, cortical plate adaptation, bone protection system (BPS)

## Abstract

**Background:** Adult patients with a thin buccal cortical plate and fragile periodontal phenotype are at high risk of dehiscence, fenestration and recession during transverse orthodontic expansion. Conventional mechanics often create a cervical compression-dominant environment that exceeds the adaptive capacity of the periodontal ligament (PDL)–bone complex. **Objectives:** This study proposes an integrative mechanobiological model in which a skeletal-anchorage-assisted loading protocol (Bone Protection System, BPS) transforms expansion into a tension-dominant regime that favours buccal phenotype preservation. **Methods:** Patient-specific finite element models were used to compare conventional expansion with a BPS-modified force system. Regional PDL stress patterns and crown/apex displacement vectors were analysed to distinguish tipping-dominant from translation-dominated mechanics. A pilot CBCT proof-of-concept (n = 1 thin-phenotype adult) with voxel-based registration quantified changes in maxillary and mandibular alveolar ridge width and buccal cortical plate thickness before and after BPS-assisted expansion. The mechanical findings were integrated with current evidence on compression- versus tension-driven inflammatory and osteogenic pathways in the PDL and cortical bone. **Results:** FEM demonstrated that conventional expansion concentrates high cervical compressive stress along the buccal PDL and cortical surface, accompanied by bending-like crown–root divergence. In contrast, the BPS protocol redirected forces to create a buccal tensile-favourable region and a more parallel crown–apex displacement pattern, indicative of translation-dominated movement. In the proof-of-concept (n = 1) CBCT case, BPS-assisted expansion was associated with preservation or increase of buccal ridge dimensions without radiographic signs of cortical breakdown. **Conclusions:** A tension-dominant orthodontic loading environment generated by a skeletal-anchorage-assisted force system may support buccal cortical preservation and vestibular phenotype reinforcement in thin-phenotype patients. The proposed mechanobiological model links these imaging and FEM findings to known molecular pathways of inflammation, angiogenesis and osteogenesis. It suggests a functional biomaterial-based strategy for widening the biological envelope of safe tooth movement.

## 1. Introduction

Orthodontic expansion in adults with a thin gingival phenotype and a narrow buccal cortical plate represents one of the most biologically vulnerable scenarios in contemporary orthodontics, as these anatomic constraints substantially reduce the safety margin for labial or buccal tooth movement [1,2,3,4,5,6,7]. The thin periodontal phenotype is characterized by reduced gingival thickness, a narrow zone of keratinized tissue and a correspondingly thin buccal alveolar plate. Clinically, the marginal tissues appear delicate and often translucent, and CBCT studies have shown a higher prevalence of buccal dehiscences, fenestrations and recession in such morphotypes under orthodontic and restorative loading. In this context, even small amounts of buccal tooth movement may exceed the adaptive capacity of the buccal plate and soft tissues, particularly when tooth displacement occurs through uncontrolled tipping rather than controlled translation [8,9]. When orthodontic forces are applied in such reduced morphologic envelopes, the periodontal ligament (PDL) readily enters a compression-dominant state, triggering hyalinization, vascular constriction and the upregulation of pro-resorptive pathways—including RANKL-mediated osteoclastogenesis and cytokine amplification. These mechanobiological responses are well documented under orthodontic loading and position, with sustained PDL compression as a primary driver of marginal bone loss and soft-tissue destabilization [10,11,12,13,14,15].

Importantly, in thin-phenotype patients, the adaptive threshold of the buccal plate is lower due to reduced cortical thickness, diminished vascular support and higher susceptibility to microdamage, which explains why labial movement within narrow alveolar housing so often results in buccal bone thinning, dehiscence, fenestration and gingival recession even under light forces [1,7,16,17].

From a mechanobiological perspective, this failure pattern is expected. Compression favors osteoclastogenesis, inflammation and tissue breakdown, whereas tensile strain stimulates angiogenesis, collagen matrix production and osteoblastic differentiation—key processes in regenerative bone remodeling [10,11,18,19,20,21,22].

Despite growing recognition of the biological fragility of thin-phenotype patients, current adjunctive procedures—such as corticotomy, PAOO and piezocision—do not reliably control root trajectory or reduce cervical compressive overload. While these methods can accelerate turnover and temporarily increase bone plasticity, their ability to protect the buccal plate from destructive stress remains limited [23,24]. Thus, the biological envelope of safe expansion remains narrow and easily violated, particularly during transverse movement.

Recent advances in finite element modeling (FEM) have provided new insights into the biomechanical determinants of buccal tissue failure. Patient-specific FEM studies demonstrate that conventional expansion frequently generates cervical pressures above the physiological tolerance threshold, while movement patterns dominated by tipping exacerbate stress concentration [25,26,27,28,29]. These analyses highlight the need for a biomechanical system capable of reducing cervical compression and inducing controlled tensile microstrain in the buccal PDL—conditions known to promote angiogenesis and osteogenesis rather than resorption [30,31,32].

Preliminary clinical observations similarly suggest that when expansion is performed under a tensile-dominant environment, the buccal phenotype may adapt more favorably than previously believed [33,34,35]. Instead of thinning or recession, some patients demonstrate increased soft-tissue thickness, improved contour convexity and enhanced cortical stability [33,34,35,36,37] -responses that stand in contrast to the well-documented compressive failure patterns described in the literature. Together with FEM evidence, these findings raise the possibility that controlled tensile strain may widen the biological limits of safe buccal movement [10,11,25,29,32,38,39,40,41].

However, no integrative mechanobiological model currently exists to explain how tensile-driven loading might reinforce both soft and hard tissues during expansion. The cellular responses of the PDL, the vascular dynamics of the buccal phenotype, the behavior of fibroblasts under tensile strain, and the cortical remodeling sequence have never been synthesized into a unified framework relevant to orthodontic expansion [10,32,40].

The objective of this work is therefore to develop a comprehensive mechanobiological model explaining how controlled tensile microstrain could promote vestibular phenotype reinforcement and buccal cortical preservation during orthodontic expansion. By integrating high-resolution FEM findings, clinical observations and established biological principles of tension-driven remodeling, this framework aims to redefine the biological envelope of safe tooth movement in anatomically vulnerable patients [32,40].

## 2. Finite Element Component of the Model

**Figure 1:**
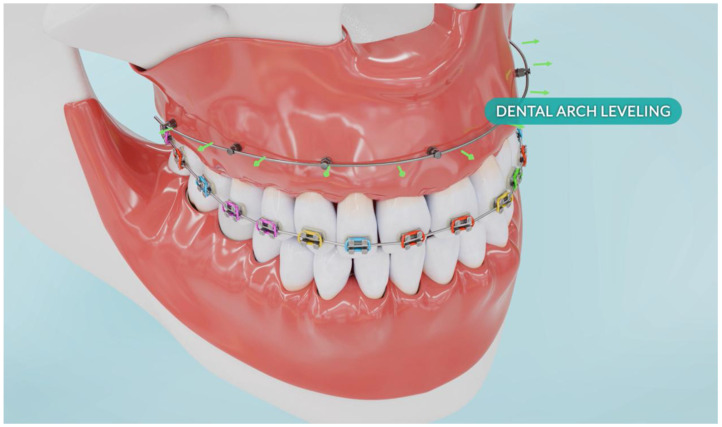
Conceptual 3D illustration of the Bone Protection System (BPS)—skeletal anchorage and controlled buccal force vector generate a tensile-dominant loading environment around the root, reducing cervical compression and promoting axial displacement. Reprinted from Ref. [42].

To mechanobiologically ground the proposed model, we incorporated a high-resolution finite element (FEM) component based on a patient-specific maxillary incisor model [23]. The numerical framework was built from CBCT-derived 3D geometries of cortical and cancellous bone, teeth and periodontal ligament (PDL), with a hyperelastic Ogden constitutive law for the PDL and density-dependent elastic properties for cortical bone. Three clinically relevant loading scenarios were analysed: (1) conventional expansion with round NiTi archwires and no corticotomy, (2) alignment with corticotomy alone, and (3) the full Bone Protection System (BPS) protocol combining deep corticotomy with skeletal anchorage and a buccal force vector. For each scenario, we evaluated buccolingual displacements of cervical and apical tracking nodes and the spatial distribution of compressive and tensile stresses within the buccal PDL and cortical plate. In all loading scenarios, a single clinically plausible transverse buccal force of approximately 1 N per tooth (≈100 g) was applied at the crown level, without active torque expression. This simplified load configuration was chosen to mimic the clinical situation during the round NiTi leveling phase, when most transverse expansion occurs, and torque control is limited. The same nominal force magnitude could also represent clear-aligner protocols delivering primarily transverse forces with minimal torque control. The conceptual biomechanical configuration is illustrated in Figure 1.

FEM demonstrated that conventional mechanics and corticotomy-only mechanics produced a tipping-dominated movement pattern: buccal crown displacement clearly exceeded apical displacement, and the cervical and apical tracking nodes frequently moved in opposite directions. In contrast, with BPS, both nodes displaced buccally and of comparable order of magnitude, indicating a translation-dominated movement of the root within the alveolar housing rather than uncontrolled tipping.

In absolute terms, crown displacement still slightly exceeded apical displacement, which is biomechanically expected because the crown emerges into the oral cavity while the root is constrained by the surrounding alveolar housing. The key qualitative change under BPS is that both nodes move buccally in the same direction, with a much smaller crown–apex discrepancy, instead of the opposite-direction pattern characteristic of uncontrolled tipping.

The most clinically relevant FEM finding, however, concerned the location of peak compressive stresses. Under conventional expansion, a continuous band of high compression was concentrated in the marginal buccal PDL and along the buccal cortical surface—precisely in the region most vulnerable to dehiscence and recession. Corticotomy alone increased overall displacement but did not substantially alter this compression belt. With BPS, the same high compressive values were redistributed away from the marginal periodontium: the buccal cervical PDL and buccal cortical surface were largely off-loaded, while zones of maximum compression shifted deeper within the alveolar housing. At the same time, a continuous tensile zone appeared along the buccal aspect of the root, replacing the previous compression-dominant pattern at the marginal level. Thus, BPS did not simply attenuate global stress extremes, but fundamentally changed where those stresses occurred—off-loading the buccal marginal tissues and creating a tension-favourable environment along the buccal root surface (Table 1 and Table 2).

## 3. Clinical Observations

The importance of precise three-dimensional anatomical evaluation has been highlighted in recent CBCT studies, including detailed assessments of the incisive canal relevant to orthodontic treatment planning [43].

Pre- and post-treatment CBCT volumes were acquired and rigidly registered to reproduce identical cross-sections for measurements. To determine the increase in bone tissue during treatment using BPS, an analysis and measurement of tissue thickness were performed on two CBCT scans acquired with identical parameters on the same device. Two sets of DICOM files of the same patient, obtained with a long time interval between them (14 months with a 2-month period for diagnosis and planning), were analyzed. In both scans, the position of the head relative to the device differed, and additionally, during the course of treatment, the geometry and structure of the jaw changed. For this reason, it was not possible to define the planes for measuring bone tissue thickness based on slice numbers; these were treated solely as auxiliary orientation information indicating the approximate region of the skull. The selection of pairs of slices for comparison was performed based on characteristic anatomical structures whose geometry was not affected by the treatment process. The matching was carried out by analyzing the geometry of reproducible structures, such as, among others, the mental foramen and the shape and size of individual teeth. Slices were considered corresponding when the shape and relative position of these structures were as similar as possible.

**Figure 2:**
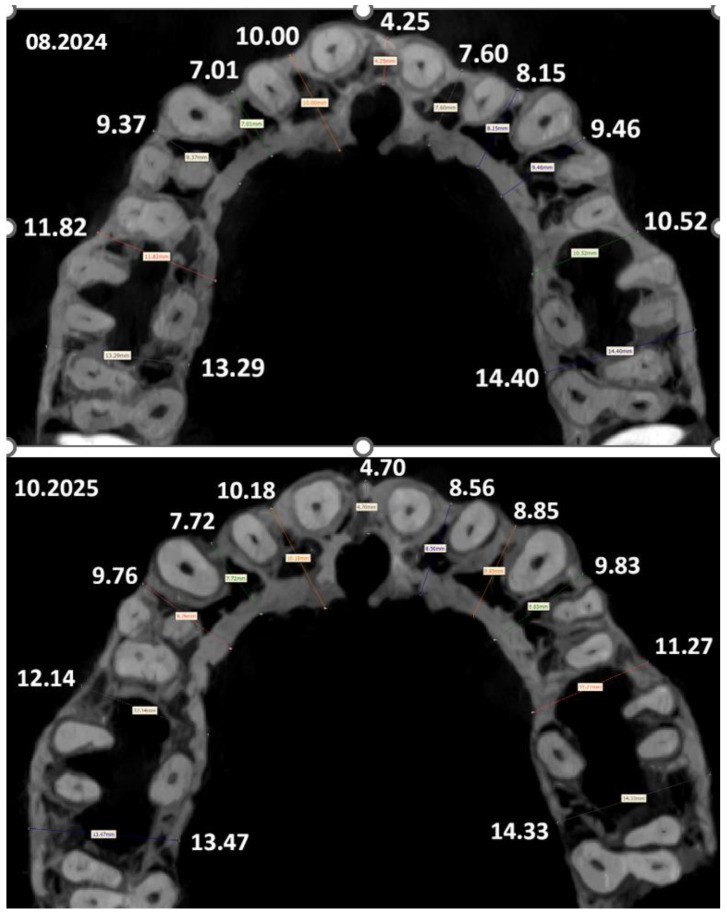
Maxillary alveolar ridge width before and after treatment (CBCT).

**Figure 3:**
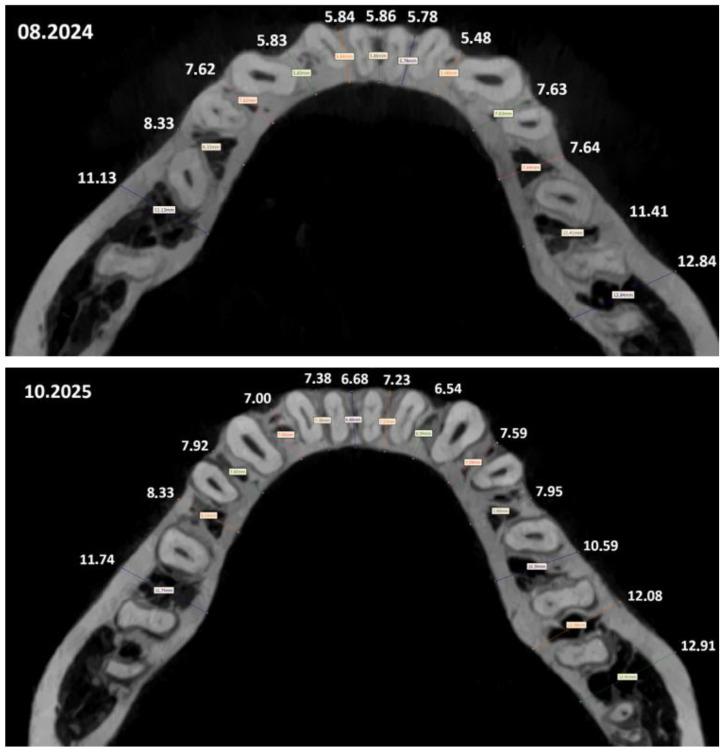
Mandibulae alveolar ridge width before and after treatment (CBCT).

Alveolar ridge width was defined as the bucco-palatal distance between the outer cortical plates at mid-root level; buccal cortical thickness was measured as the perpendicular distance from the root surface to the buccal cortex. Measurements were repeated twice by a calibrated examiner (ICC > 0.92; Dahlberg error < 0.2 mm). Quantitative outcomes are reported in Table 3 and Table 4, with representative cross-sections provided in Figure 2 and Figure 3.

## 4. Mechanobiological Model

The proposed mechanobiological model delineates how controlled tensile microstrain, achieved through optimized biomechanics, may drive a regenerative rather than destructive periodontal response during orthodontic expansion. This multilevel model integrates (1) the biomechanical redistribution of forces revealed in finite element simulations, (2) the cellular and molecular reactions of the periodontal ligament (PDL) to tension and compression, and (3) the tissue-level adaptations observed clinically. It is important to emphasize that the finite element load configuration was defined at the tooth level and did not depend on a specific bracket prescription or ligation mode. During the clinically relevant leveling phase with round NiTi archwires, neither conventional nor self-ligating brackets are able to fully prevent uncontrolled tipping in thin-phenotype patients, because torque expression is intrinsically limited. The BPS protocol modifies the force system at the root level by combining skeletal anchorage with a controlled buccal vector, and can therefore be integrated with different fixed-appliance systems—or even clear aligners—provided that the net buccal force magnitude is comparable.

1. Biomechanical Level: From Cervical Compression to Tension

Finite element studies have repeatedly demonstrated that conventional expansion results in high cervical compressive peaks—even in low-force protocols—exceeding the physiological load tolerance of the PDL and microvascular network [25,29,38]. These stresses correspond to the mechanobiological threshold at which the PDL shifts into hypoxia, hyalinization, and osteoclastic activation [10,22]—Figure 2.

**Figure 4:**
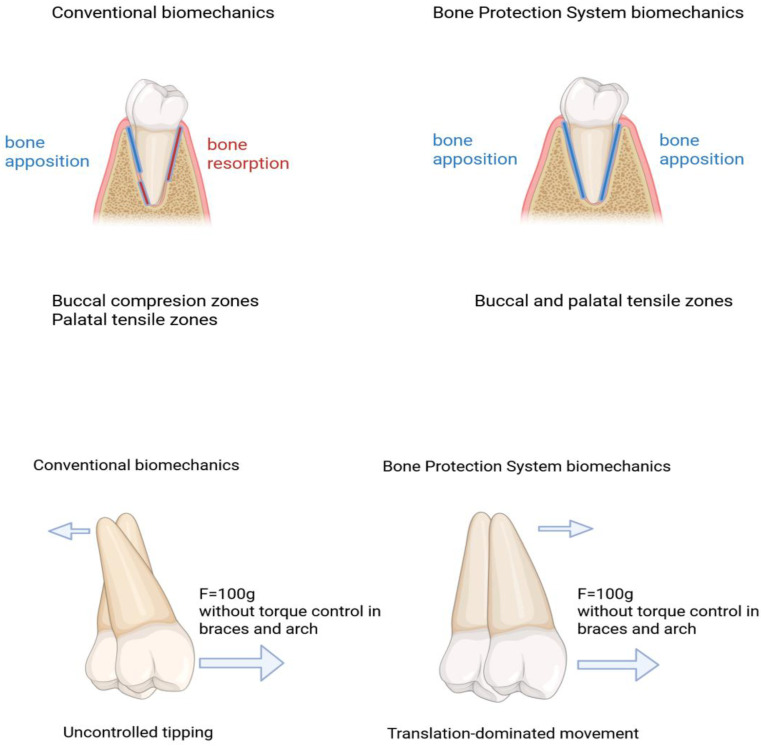
Biomechanical Stress Map: Compression vs Tension in PDL and displacement vector fields illustrating tipping—dominant versus translation-dominant mechanics (Classical vs Optimized Mechanics).

In contrast, controlled biomechanics that promote axial, translation-dominated movement alter the local stress field in a qualitatively different way: cervical compression is no longer concentrated in the marginal buccal PDL but is redistributed deeper within the alveolar housing, the stress field along the buccal root surface becomes more uniform, with relative off-loading of the buccal cervical PDL and cortical plate, and a buccal tensile zone emerges along the root surface, within a microstrain range that has been associated with osteogenic and angiogenic activation [44,45]—Figure 4.

This mechanical environment aligns with classical mechanotransduction data showing that tension, unlike compression, maintains capillary perfusion, permits cellular signaling, and recruits reparative pathways [10,22,46].

Thus, the first pillar of the model is the biomechanical transformation:

FROM: compression-dominant stress → tipping → cervical overload

TO: tensile-dominant stress → translational root movement → vascular patency.

2. PDL-Level Mechanobiology: Divergent Signaling in Tension vs Compression

Compression-side biology (pathologic):

Compression activates RANKL, TNF-α, IL-1β, prostaglandins, and increases osteoclastic differentiation while locally inhibiting angiogenesis [1,10,22,47,48,49]. These molecular events explain the high incidence of buccal bone loss, dehiscence, and gingival recession during uncontrolled expansion in thin phenotypes.

Tension-side biology (regenerative):

Tensile strain triggers a completely different biological program:

- Upregulation of COL1A1 and extracellular matrix production [12,50].
- Runx2 and Osterix activation initiating osteoblastic commitment [12,13,14,15,16,17,18,19,20,21,22,23,24,25,26,27,28,29,30,31,32,33,34,35,36,37,38,39,40,41,43,44,45,46,47,48,49,50].
- VEGF and HIF-1α induction enabling angiogenesis and improved perfusion [51,52,53].
- Wnt/β-catenin pathway activation crucial for osteogenic lineage stabilization [44,54,55].

Together, these data support the concept that tensile microstrain switches the PDL into an anabolic state, favoring bone formation rather than resorption.

3. Cortical-Level Biology: Tension-Driven Apposition and Reduced Resorption

The cortical bone response under tensile forces resembles the principles seen in distraction osteogenesis:

- Latency phase: Recruitment of mesenchymal stem cells (MSCs) and TGF-β signaling [56].
- Activation phase: MAPK/ERK and BMP-2/Smad pathways enhancing osteoblast differentiation [57,58,59,60,61,62,63].
- Consolidation phase: Maturation of osteoid with osteocalcin and osteopontin expression [64,65].

In contrast, compression has been shown to accelerate cortical porosity, reduce vascular supply, and potentiate osteoclastic catabolism—changes particularly detrimental in thin buccal bone [1,10,22,49].

The model therefore predicts that when expansion occurs under tensile microstrain, the buccal cortex may undergo controlled apposition rather than thinning, a hypothesis consistent with early clinical observations in thin-biotype patients treated with tension-oriented mechanics.

4. Load Trajectory and Displacement Pattern (Tipping vs Translation)

A critical element of the proposed mechanobiological framework is the trajectory of structural displacement generated under different loading configurations. Conventional transverse loading produces a bending-dominant moment, resulting in divergence of coronal and apical displacement vectors. This “tipping-like” pattern generates steep compressive gradients in the cervical PDL, limiting the extent and uniformity of tensile microstrain [25,29,38,46].

In contrast, the BPS configuration redirects load in a manner that aligns displacement vectors along a common axis, creating a translation-dominant pattern. This reduces bending moments and produces a more homogenous tensile stress field surrounding the root surface. Such displacement coherence is essential for sustaining angiogenic and osteogenic signaling and may explain the clinical phenomenon of phenotype preservation despite transverse displacement.

This distinction between bending-dominant and translation-dominant loading is illustrated in Figure 4.

5. Soft-Tissue Level: Tensile Stimulus and Phenotype Reinforcement

The buccal gingival phenotype—traditionally considered fragile and prone to recession—is biomechanically sensitive to the direction of PDL stress. While compressive forces can compromise the soft-tissue envelope, tensile environments have been associated with fibroblast activation, increased production of collagen types I and III, and remodeling of the extracellular matrix [66,67,68,69]. Such responses may contribute to the thickening and stabilization of the supracrestal connective tissue, as well as improved vascularization, providing a plausible mechanism by which soft tissues can maintain or reinforce their phenotype despite orthodontic tooth movement.

This concept constitutes the fourth pillar of the proposed mechanobiological model: tension → fibroblast activation → matrix reinforcement → phenotype stabilization [66,67,68,69].

6. Integrated Tissue Response: A Unified Mechanobiological Loop

**Figure 5:**
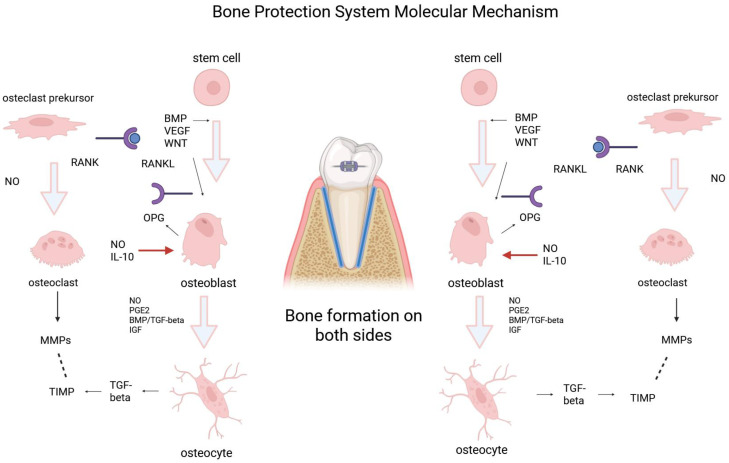
Tensile-Driven Mechanobiological Pathway—integrated model linking tensile stress to angiogenesis, PDL matrix synthesis, osteogenic activation and cortical bone apposition.

The proposed “Tensile-Driven Buccal Reinforcement Model” rests on five coordinated events:

1. Biomechanical redistribution: Reduction in compression, emergence of tensile stress - The FEM component of this study demonstrated that force-vector optimization can reduce cervical compression and generate a buccal tensile zone within a microstrain range associated with osteogenic activation. These findings are consistent with previously published FEM analyses showing that conventional expansion produces localized cervical overload, whereas translational mechanics substantially modify stress distribution [25,70].
2. PDL tension signaling: VEGF, COL1A1, Runx2, OPN pathways → anabolic switch [71,72,73,74,75]—Figure 5.
3. Angiogenic activation: Improved perfusion enables remodeling rather than resorption [72]
4. Cortical adaptation: Tension-driven apposition outcompetes cortical thinning [25,72]
5. Soft-tissue reinforcement: Increased matrix deposition stabilizes the vestibular phenotype [66,67]

This integrated loop explains a clinical paradox: why thin-biotype patients, typically at the highest risk of recession and dehiscence, may exhibit reinforcement instead of breakdown when expansion is performed under tensile-dominant mechanics.

This integrated loop provides a mechanistic framework explaining why thin-biotype patients, typically at higher risk of recession and dehiscence, might resist tissue breakdown under tensile-dominant orthodontic expansion. It is important to note that while the osteogenic and angiogenic components (points 2–4) are supported by experimental and modeling evidence, the soft-tissue reinforcement aspect (point 5) is currently hypothetical and requires further clinical validation.

7. Clinical Implication of the Model

The model suggests that the key determinant of periodontal safety during expansion is not the magnitude of force, but the quality of the biomechanical environment—specifically, whether cervical tissues experience compression (pathologic) or tension (regenerative).

It implies that the “biological limit of tooth movement” is dynamic and can be expanded safely if tensile microstrain is maintained within the PDL and the buccal cortex [25,29,38].

## 5. Discussion

Orthodontic expansion performed within the limits of thin buccal alveolar housing has long been associated with cortical thinning, dehiscence formation and gingival recession. These complications are fundamentally rooted in the biological response of the periodontal ligament (PDL) to cervical compressive stresses that exceed its adaptive capacity. Classical mechanobiological studies have demonstrated that compression activates a cascade of destructive molecular events, including RANKL upregulation, pro-inflammatory cytokine release and osteoclastic differentiation [10,22]. This shift in signaling leads to marginal bone breakdown and tissue vulnerability, particularly in thin-phenotype individuals whose buccal plate volume and vascular reserve are inherently limited [1,49].

In contrast, tensile strain initiates a predominantly regenerative response. Tension preserves vascular perfusion and increases the expression of osteogenic and angiogenic markers such as COL1A1, osteopontin, osteocalcin and Runx2 [72,76,77,78]. It also activates the Wnt/β-catenin pathway, a central regulator of mesenchymal stem cell osteogenic differentiation [44], and induces VEGF- and HIF-1α-dependent angiogenesis essential for tissue survival and remodeling [53,77]. These processes collectively explain why tensile environments support bone apposition, collagen deposition and structural reinforcement and help to account for the divergent outcomes observed clinically during expansion. CBCT studies have consistently shown that conventional transverse expansion—driven primarily by uncontrolled tipping—results in cervical compression, buccal bone thinning and fenestrations [1,49,79,80,81,82,83,84]. This occurs even under low-force conditions, underscoring the dominant role of force direction rather than magnitude in determining biological risk.

Findings from distraction osteogenesis provide an important parallel for interpreting tension-driven periodontal adaptation. In distraction models, controlled strain induces a coordinated sequence of events: recruitment of mesenchymal stem cells, activation of TGF-β and BMP-2 pathways, MAPK/ERK signaling and progressive collagen matrix deposition [85,86,87,88,89,90,91]. Similar pathways are activated in tension-loaded PDL cells during orthodontic movement. These converging data suggest that tensile microstrain is a potent driver of osteogenesis and angiogenesis across different tissues and loading conditions [59,63,73,92,93,94]. FEM-based observations provide critical insights into the biomechanical prerequisites for tension-driven regeneration. Recent patient-specific simulations have demonstrated that conventional expansion produces localized compressive peaks well above physiological tolerance, whereas force systems that promote axial translation generate a buccal tensile zone within the biologically favorable microstrain range [25,70]. This aligns with established experimental thresholds indicating that high compression impairs cell viability and vascular flow, while moderate tension supports anabolic metabolism and matrix synthesis [95].

Within this context, the FEM component of the present work is critical because it demonstrates that BPS does not merely fine-tune conventional mechanics but creates a biomechanical regime that was previously unattainable in thin-phenotype patients. By converting uncontrolled tipping into translation-dominated movement while simultaneously off-loading the marginal buccal PDL and cortical surface, BPS establishes a tensile-favourable environment exactly where conventional expansion generates destructive compression. This combination—translation with round NiTi wires and marginal buccal tension instead of compression—provides a mechanistic bridge between the favourable clinical outcomes observed in BPS-treated cases and the well-established biology of tension-driven angiogenesis and bone apposition [96].

Taken together, these data suggest that the biological envelope of safe expansion is not fixed but instead depends on the mechanical environment experienced by the PDL and surrounding cortical bone. When expansion is performed under cervical compression, tissue breakdown is the expected outcome; when performed under controlled tensile stress, adaptive reinforcement becomes possible. This theoretical framework provides a biologically plausible explanation for clinical observations in which thin-biotype patients treated with tension-favorable mechanics exhibit soft-tissue thickening, improved gingival margin stability and preservation of the buccal cortex—responses traditionally considered unattainable without grafting.

The proposed mechanobiological model integrates these observations into a unified concept:

Tensile stress enables periodontal regeneration by simultaneously maintaining vascular patency, inhibiting destructive inflammatory cascades and activating osteogenic and angiogenic pathways. This dual effect—biomechanical and biological—may explain the unexpected reinforcement of the buccal phenotype reported under tensile-driven conditions [50,97,98,99].

Importantly, this framework also clarifies why corticotomy alone cannot fully protect thin buccal structures. Although corticotomy induces a transient increase in remodeling potential through the regional acceleratory phenomenon (RAP), it does not alter the direction of tooth movement or the cervical stress field. As a result, compressive overload and the associated risk of resorption persist even when RAP is activated [23,24,100,101,102,103]. This distinction underscores the need for force-vector control—not merely bone turnover—to achieve biologically safe expansion. It is important to note that, in the present manuscript, soft-tissue reinforcement remains a mechanobiological hypothesis. While the proposed pathways and FEM-based loading conditions are biologically plausible, the actual gingival thickness and contour changes were not quantitatively evaluated here and therefore require confirmation in dedicated clinical studies.

Direct histologic confirmation of the underlying tissue responses and real-time assessment of perfusion were not feasible in a clinical human study. As such, the mechanobiological inferences presented here are based on structural imaging (CBCT) and FEM-derived loading patterns, which are consistent with established biological responses to tensile stress but do not directly visualize cellular or vascular dynamics. Future studies incorporating advanced perfusion imaging or translational animal models will be essential to validate these mechanisms.

### Limitations

Several limitations must be acknowledged. While the mechanobiological principles underlying tension-mediated reinforcement are well established in periodontal and orthopedic biology, clinical evidence in orthodontics remains limited and requires larger, controlled studies. Furthermore, the optimal magnitude, duration and spatial distribution of tensile stress necessary to induce predictable regeneration are not yet fully defined. Future work should incorporate advanced imaging, volumetric soft-tissue analyses and long-term follow-up to validate the model’s clinical implications. The clinical CBCT component represents a pilot, proof-of-concept dataset designed to illustrate the mechanobiological implications of tensile-dominant loading rather than to determine generalized clinical outcomes. The CBCT findings should therefore be interpreted as preliminary, hypothesis-generating evidence supporting the proposed model and requiring validation in larger controlled cohorts. Accordingly, the findings should be interpreted as preliminary evidence supporting the proposed model, requiring validation in larger controlled cohorts.

From a practical standpoint, BPS also has clear limitations. The protocol requires temporary anchorage devices and corticotomy, which increase cost, chair time and technique sensitivity. In addition, by shifting stresses deeper into the alveolar housing, BPS may theoretically increase the risk of deeper root resorption or trabecular microdamage, even if cervical compression is reduced. These potential adverse effects were not observed in the present proof-of-concept case but must be monitored in future controlled cohorts.

From a biomechanical standpoint, the intention of BPS is to redirect the load so that tensile elongation occurs predominantly within the periodontal ligament and within the corticotomised buccal cortical segments. By pulling on mini-implants placed in the buccal bone segments, the protocol produces controlled stretching of the corticotomised plate rather than uncontrolled crown tipping against an intact, thin cortex. However, if excessive forces were applied, the same mechanism could, in theory, overload the PDL fibres and the weakened cortical segment. For this reason, careful force calculation, light transverse loading and regular radiographic and clinical monitoring are essential components of the protocol, and BPS should be restricted to carefully selected high-risk patients until larger prospective datasets become available.

Despite these limitations, the mechanobiological framework presented here offers a new conceptual foundation for understanding how controlled tensile stress/microstrain may expand the biological limits of orthodontic tooth movement in thin-phenotype patients. By integrating FEM insights, cellular biology and clinical observations, this model provides a biologically grounded rationale for designing safer and more predictable expansion strategies in anatomically vulnerable populations.

**Clinical Relevance:** Thin buccal cortical bone and a delicate gingival phenotype significantly increase the risk of dehiscence, fenestration and recession during orthodontic expansion. This mechanobiological model shows that the direction of force—not its magnitude—is the primary determinant of periodontal response. By promoting tensile rather than compressive loading of the PDL, clinicians may create a biologically favorable environment that supports angiogenesis, bone apposition and soft-tissue stability. These insights provide a scientific basis for safer expansion strategies in high-risk patients and may broaden the limits of predictable orthodontic treatment.

## 6. Conclusions

This work proposes a mechanobiological framework explaining how controlled tensile stress/microstrain may enable periodontal reinforcement during orthodontic expansion in thin-biotype patients. By integrating finite element evidence, established pathways of tension-mediated remodeling and a single proof-of-concept CBCT case, the model supports the view that the mechanical environment—rather than force magnitude—is the determining factor governing tissue adaptation. When the PDL is redirected from cervical compression toward biologically favorable tensile stress, vascular patency is preserved, osteogenic and angiogenic cascades are activated, and both soft and hard tissues undergo adaptive reinforcement rather than breakdown. These insights may redefine the biological envelope of safe orthodontic treatment and offer a foundation for developing expansion protocols that prioritize periodontal safety in anatomically vulnerable individuals.

## Figures and Tables

**Table 1:** Qualitative FEM comparison of tooth movement patterns and marginal buccal loading for conventional mechanics, corticotomy alone, and the Bone Protection System (BPS).

Parameter	Conventional Mechanics	Corticotomy Alone	BPS Mechanics
**Crown–root movement pattern**	Tipping-dominated (crown ≫ apex; cervical and apical nodes often in opposite directions)	Tipping-dominated (greater overall displacement, but same tipping pattern)	Translation-dominated (crown and apex move buccally in the same direction with reduced crown–apex discrepancy)
**Marginal buccal PDL loading**	Continuous band of high compression along the cervical third	Continuous band of high compression along the cervical third	Marginal buccal PDL largely off-loaded or in low-load / mild tensile range
**Buccal cortical surface (cervical region)**	Heavily loaded, compression-dominant	Heavily loaded, compression-dominant	Off-loaded compared with conventional; no continuous cervical compression belt
**Location of peak compressive stresses**	Concentrated at marginal buccal PDL and buccal cortical plate	Concentrated at marginal buccal PDL and buccal cortical plate	Shifted deeper within the alveolar housing, away from the thin buccal plate
**Buccal tensile zone on expansion side**	Absent	Absent	Present; continuous tensile band along the buccal aspect of the root

**Table 2:** End-point crown and apex displacements (µm).

Scenario (Figure)	E (GPa)	Crown Disp. at t = 1.0 (µm)	Apex Disp. at t = 1.0 (µm)	Pattern
No corticotomy	12.5	+2.25	−0.36	tipping-dominant (opposite sign)
No corticotomy	27.5	+1.75	−0.34	tipping-dominant (opposite sign)
3 mm corticotomy	12.5	+2.85	−0.30	tipping-dominant (opposite sign)
3 mm corticotomy	27.5	+2.10	−0.30	tipping-dominant (opposite sign)
BPS	12.5	+1.60	+0.70	translation-dominant (same sign)
BPS	27.5	+2.00	+0.20	translation-dominant (same sign)

**Table 3:** Maxillary and Mandibular Alveolar Ridge Width (CBCT).

**Maxilla**			
Segment	August 2024 (mm)	October 2025 (mm)	Change (mm)
1	11.82	12.14	+0.32
2	9.37	9.76	+0.39
3	7.01	7.72	+0.71
4	10.00	10.18	+0.18
5	4.25	4.70	+0.45
6	7.60	8.56	+0.96
7	8.15	8.85	+0.70
8	9.46	9.83	+0.37
9	10.52	11.27	+0.75
10	14.40	14.33	−0.07
11	13.29	13.47	+0.18
**Mandibulae**			
Segment	August 2024 (mm)	October 2025 (mm)	Change (mm)
1	11.13	11.74	+0.61
2	8.33	8.33	0.00
3	7.62	7.92	+0.30
4	5.83	7.00	+1.17
5	5.84	7.38	+1.54
6	5.86	6.68	+0.82
7	5.78	7.23	+1.45
8	5.48	6.54	+1.06
9	7.63	7.59	−0.04
10	7.64	7.95	+0.31
11	11.41	10.59	−0.82
12	12.84	12.08	−0.76

**Table 4:** Maxilla buccal plate change before and after treatment (CBCT).

No.	Pre (mm)	Post (mm)	Δ (Post–Pre) (mm)
1	1.07	1.03	−0.04
2	0.87	1.03	0.16
3	0.45	0.53	0.08
4	0.38	1.05	0.67
5	0.18	0.42	0.24
6	0.31	0.48	0.17
7	0.30	0.47	0.17
8	0.50	0.75	0.25
9	0.77	1.07	0.30
10	0.33	0.57	0.24
11	0.60	0.70	0.10
12	0.25	0.41	0.16
13	0.38	0.81	0.43
14	0.37	0.61	0.24
15	0.80	1.35	0.55
16	1.11	1.28	0.17

## Data Availability

De-identified data supporting the findings of this study are available from the corresponding author upon reasonable request. Patient-level CBCT DICOM data are not publicly available due to privacy and ethical restrictions.
