# Correction: Kuc et al. Tension-Dominant Orthodontic Loading and Buccal Periodontal Phenotype Preservation: An Integrative Mechanobiological Model Supported by FEM and a Proof-of-Concept CBCT. *J. Funct. Biomater.* 2026, *17*, 47

**DOI:** 10.3390/jfb17060259

**Published:** 2026-05-22

**Authors:** Anna Ewa Kuc, Jacek Kotuła, Kamil Sybilski, Szymon Saternus, Jerzy Małachowski, Natalia Kuc, Grzegorz Hajduk, Joanna Lis, Beata Kawala, Michał Sarul, Magdalena Sulewska

**Affiliations:** 1 Department of Dentofacial Orthopedics and Orthodontics, Wroclaw Medical University, 50-425 Wroclaw, Poland; j_kotula@poczta.onet.pl (J.K.); joanna.lis@umw.edu.pl (J.L.); beata.kawala@umw.edu.pl (B.K.); 2 Faculty of Mechanical Engineering, Military University of Technology, 00-908 Warsaw, Poland; kamil.sybilski@wat.edu.pl (K.S.); szymon.saternus@wat.edu.pl (S.S.); jerzy.malachowski@wat.edu.pl (J.M.); 3 Faculty of Medicine, Medical University in Bialystok, ul. Kilińskiego 1, 15-089 Bialystok, Poland; nataliakuc.med@gmail.com; 4 Chair and Department of Oral Surgery, Medical University of Lublin, Witolda Chodźki 6 Street, 20-093 Lublin, Poland; 5 Department of Integrated Dentistry, Wroclaw Medical University, 50-425 Wroclaw, Poland; michal.sarul@umw.edu.pl; 6 Department of Periodontal and Oral Mucosa Diseases, Medical University in Bialystok, ul. Waszyngtona 13, 15-269 Bialystok, Poland; magdalena.sulewska@umb.edu.pl

## Missing Citation

In the original publication [1], reference [42] was not cited in Figure 1. The citation has now been inserted in Figure 1 and should read:

**Figure 1.** Conceptual 3D illustration of the Bone Protection System (BPS)—skeletal anchorage and controlled buccal force vector generate a tensile-dominant loading environment around the root, reducing cervical compression and promoting axial displacement. Reprinted from Ref. [42].

## Reference

The corresponding reference has been added to the References list as follows:

42. Kuc, A.E.; Sulewska, M.; Sybilski, K.; Kotuła, J.; Hajduk, G.; Saternus, S.; Małachowski, J.; Bar, J.; Lis, J.; Kawala, B.; et al. Mechanobiological Regulation of Alveolar Bone Remodeling: A Finite Element Study and Molecular Pathway Interpretation. *Biomolecules* **2026**, *16*, 150. https://doi.org/10.3390/biom16010150.

With this correction, the order of some references has been adjusted accordingly. The authors state that the scientific conclusions are unaffected. This correction was approved by the Academic Editor. The original publication has also been updated.
